# Mr. Leese's Case of Stricture of the Urethra

**Published:** 1806-08

**Authors:** Ed. Leese

**Affiliations:** Duke-street, Manchester-square


### To Dr. BATTY.

SIR,

THE following case, with its dissection, appears to me to differ in some points from those generally to be met with either in practice, or as described by authors; if you think it may be of any use to others, and will cause it to be inserted in that very useful publication, the Medical and Physical Journal, you will oblige,

Yours, respectfully,

ED. LEESE.

*Duke-street, Manchester-square,*

11 *July*, 1806.

I. B. a hair-dresser, aged 57, for the last fifteen years of his life had been much troubled with pain and difficulty in voiding urine; he generally lived temperately, but in his earlier days had gonorrhœa, which, I understand, had been removed for some time before these complaints became troublesome; but how long I do not know. For the obstruction he had been advised diuretics, bougies, &c. by which he received little if any benefit; it had been suspected there was stone in the bladder, and he was accordingly sounded by the direction, and in the presence of some respectable practitioners; no calculus was discovered. His complaints were not removed, though he remained a long time under their direction. About six or eight months before his death, he was a considerable time under the superintendance of a respectable hospital surgeon, who, I was informed, made frequent use of caustic bougies, &c. without producing any relief; at length he became so ill as to be confined to the house, and about a fortnight before his death he came under my care. I found him very much emaciated in constitution, complaining, that although he had very frequent inclinations, he passed but little urine, and that with great difficulty, and in drops, attended with much pain; he complained also of great uneasiness in each of the lumbar regions, particularly the right; numbness in the course of the ischiatic nerves, and an irregular fever. Several attempts were made to introduce a catheter, and bougies of different sizes, but without success, excepting once, when a very small bougie passed into the bladder; on the points of some of them, when withdrawn, was to be observed an indentation, much as if they had been forced against a septum dividing the natural passage into two; this prevented them from passing more than about 6½ inches. After remaining in this state a few days, and voiding but a small quantity of urine, he died.

Permission being obtained to open the body, it was done, with the assistance of a medical friend, the next day.

On removing the parieties of the abdomen, the liver and intestines were found in a sound state; the omentum reduced to a mere membrane, not containing any fatty substance; in removing the intestines from the right side, a finger ruptured an abscess, the sac of which seemed to be a distention of the peritoneal coat of the right kidney, extending forwards and downwards over the psoas muscle, and contained full a pint of pus, of thick consistence, and without without smell. The left kidney was very much reduced in size, its texture very loose and flaccid, and with the ureter formed an immense bag, containing turbid urine to the quantity of 12 oz. as was afterwards ascertained by measuring. The openings from the ureter to the bladder were as large as a goose quill, so that any fluid as readily passed from the bladder to the ureter, as the reverse; this enlargement of the ureter and its opening, no doubt, had been produeed by the bladder always containing more or less urine, not being able to empty itself. The bladder was firmly contracted upon the urine it contained, which was about half a pint; the muscular fibres were much thickened in substance, from the great efforts they had made to overcome the obstruction; the inner coat was contracted into rugæ, and of a yellowish green tinge, but not diseased; all the parts posterior to the strictured part, such as the verumontanum, the lacunæ, &c. were enlarged, and had an appearance like leather that had been macerated in a fluid.

The prostate gland was not diseased, though enlarged like other parts near it. On opening the urethra at the strictured part behind the bulb, about 6¼ inches from the extremity, we found the natural passage so obstructed as to be almost imperforate, but at the under side was a short artificial one, about a quarter inch in length, which had doubtless been made by attempts to pass bougies, &c. but having been once formed, would render such attempts doubly difficult and uncertain. At this part we found a cellular rather firm substance on the outer side of the membrane, *not surrounding the canal,* but only on the side next the corpora carvernosa, yet compressing and altering its direction, so as to render it nearly impossible for any instrument to pass.

Stricture of the urethra has generally been defined as, " contraction of the transverse fibres of membrane forming that canal." This case was certainly different, and probably presented more difficulties than are usually to be met with, to any attempts that might have been made to relieve it.

